# Erratum: Acquired antibiotic resistance genes: an overview

**DOI:** 10.3389/fmicb.2012.00384

**Published:** 2012-11-16

**Authors:** Marilyn C. Roberts, Stefan Schwarz, Henk J. M. Aarts

**Affiliations:** ^1^Department of Environmental and Occupational Health Sciences, School of Public HealthSeattle, WA, USA; ^2^Institute of Farm Animal Genetics, Friedrich-Loeffler-InstitutNeustadt-Mariensee, Germany; ^3^National Institute of Health and the Environment (RIVM), Antonie van Leeuwenhoeklaan 9Bilthoven, Netherlands

**A commentary on**

**http://www.frontiersin.org/Antimicrobials,_Resistance_and_Chemotherapy/10.3389/fmicb.2011.00203/abstract**

**Acquired antibiotic resistance genes: an overview**

by van Hoek, A. H. A. M., Mevius, D., Guerra, B., Mullany, P., Roberts, A. P., and Aarts, H. J. M. (2011). Front. Microbio. 2:203. doi: 10.3389/fmicb.2011.00203

Dr. Marilyn C. Roberts and Dr. Stefan Schwarz have contacted the authors of the original publication with several comments and suggestions to better harmonize the correct nomenclature of the antibiotic resistance genes, as the gene names were not always correctly presented in the various tables given.

Authors often pick their own gene names which in many cases have been approved for use for other genetically distinct genes or give names to determinants which were already given an approved designated name. Therefore, we (Dr. Marilyn C. Roberts and Dr. Stefan Schwarz and Dr. Henk J. M. Aarts on behalf of the authors of the original publication) would like to present here the correct nomenclature and mechanistic features of the antibiotic resistance genes belonging to the following classes: Aminoglycosides (Table 1), Phenicols (Table 3), Macrolides–Lincosamides–Streptogramin B (Table 4), Quinolones (Table 5), Tetracyclines (Table 6), and Trimethoprim (Table 7). In addition some additional information is given on the various classes of antibiotic resistance genes as also a section regarding the antibiotic class Oxazolidinones has been added. Table 2 was correctly displayed by van Hoek et al. (2011) but has been updated.

**Table 1:** Acquired aminoglycoside resistance genes^*^.

**Mechanism**	**Gene name**	**Length (nt)**	**Accession number or reference**	**Coding region**	**Genera**
ACT	*aac(2′)-Ia*	537	L06156	264… 800	*Providencia*
	*aac(2′)-Ib*	588	U41471	265… 852	*Mycobacterium*
	*aac(2′)-Ic*	546	U72714	373… 918	*Mycobacterium*
	*aac(2′)-Id*	633	U72743	386… 1018	*Mycobacterium*
	*aac(2′)-Ie*	549	NC_011896	3039059… 3039607	*Mycobacterium*
	*aac(3)-I*	465	AJ877225	5293… 5757	*Pseudomonas*
	*aac(3)-Ia*	534	X15852	1250… 1783	*Acinetobacter*, *Escherichia*,* Klebsiella*,* Salmonella*, *Serratia*, *Streptomyces*
	*aac(3)-Ib*	531	L06157	555… 1085	*Pseudomonas*
	*aac(3)-Ib*-*aac(6′)-Ib*	1005	AF355189	1435… 2439	*Pseudomonas*
	*aac(3)-Ic*	471	AJ511268	1295… 1765	*Pseudomonas*
	*aac(3)-Id*	477	AB114632	104… 580	*Proteus*, *Pseudomonas*, *Salmonella*, *Vibrio*
	*aac(3)-Ie*	477	AY463797	8583… 9059	*Proteus*, *Pseudomonas*, *Salmonella*, *Vibrio*
	*aac(3)-If*	465	AY884051	61… 525	*Serratia*, *Pseudomonas*
	*aac(3)-Ig*	477	CP000282	2333620… 2334096	*Saccharophagus*
	*aac(3)-Ih*	459	CP000490	509912… 510370	*Paracoccus*
	*aac(3)-Ii*	459	CP000356	638262… 638720	*Sphingopyxis*
	*aac(3)-Ij*	465	CP000155	6963012… 6963476	*Hahella*
	*aac(3)-Ik*	444	BX571856	765853… 766296	*Staphylococcus*
	*aac(3)-IIa*	861	X51534	91… 951	*Acinetobacter*, *Enterobacter*, *Escherichia*,* Klebsiella*,* Pseudomonas*, *Salmonella*
	*aac(3)-IIb*	810	M97172	656… 1465	*Serratia*
	*aac(3)-IIc*	861	X54723	819… 1679	*Escherichia*
	*aac(3)-IId*	861	EU022314	1… 861	*Escherichia*
	*aac(3)-IIe*	861	EU022315	1… 861	*Escherichia*
	*aac(3)-IIIa*	816	X55652	1124… 1939	*Pseudomonas*
	*aac(3)-IIIb*	738	L06160	984… 1721	*Pseudomonas*
	*aac(3)-IIIc*	840	L06161	106… 945	*Pseudomonas*
	*aac(3)-IVa*	786	X01385	244… 1029	*Escherichia*
	*aac(3)-Va;*				
	*see aac(3)-IIa*				
	*aac(3)-Vb;*				
	*see aac(3)-IIb*				
	*aac(3)-VIa*	900	M88012	193… 1092	*Enterobacter*, *Escherichia*, *Salmonella*
	*aac(3)-VIIa*	867	M22999	493… 1359	*Streptomyces*
	*aac(3)-VIIIa*	861	M55426	466… 1326	*Streptomyces*
	*aac(3)-IXa*	846	M55427	274… 1119	*Micromonospora*
	*aac(3)-Xa*	855	AB028210	2711… 3565	*Streptomyces*
	*aac(6′)*	441	AY553333	1392… 1832	*Pseudomonas*
	*aac*	555	AJ628983	1985… 2539	*Pseudomonas*
	*aac(6′)*	402	DQ302723	81… 482	*Pseudomonas*
	*aac(6′)*	555	EU912537	2092… 2646	*Pseudomonas*
	*aac(6′)-Ia*	558	M18967	757… 1314	*Citrobacter*,* Escherichia*,* Klebsiella*, *Shigella*
	*aac(6′)-Ib*	606	M21682	380… 985	*Klebsiella*, *Proteus*, *Pseudomonas*
	*aac(6′)-Ib-cr*	519	EF636461	1124… 1642	*Enterobacter*, *Escherichia*,* Klebsiella*, *Pseudomonas*, *Salmonella*
	*aac(6′)-Ic*	441	M94066	1554… 1994	*Serratia*
	*aac(6′)-Id*	450	X12618	905… 1354	*Klebsiella*
	*aac(6′)-Ie;*				
	*see aac(6′)-aph(2″)*				
	*aac(6′)-If*	435	X55353	279… 713	*Enterobacter*
	*aac(6′)-Ig*	438	L09246	544… 981	*Acinetobacter*
	*aac(6′)-Ih*	441	L29044	352… 792	*Acinetobacter*
	*aac(6′)-Ii*	549	L12710	169… 717	*Enterococcus*
	*aac(6′)-Ij*	441	L29045	260… 700	*Acinetobacter*
	*aac(6′)-Ik*	438	L29510	369… 806	*Acinetobacter*
	*aac(6′)-Il*	522	Z54241	530… 1051	*Acinetobacter*, *Citrobacter*
	*aac(6′)-Im*	537	AF337947	1215… 1751	*Escherichia*
	*aac(6′)-In*	573	Wu et al., 1997	–	*Citrobacter*
	*aac(6′)-Iq*	552	AF047556	127… 678	*Klebsiella*, *Salmonella*
	*aac(6′)-Ir*	441	AF031326	1… 441	*Acinetobacter*
	*aac(6′)-Is*	441	AF031327	1… 441	*Acinetobacter*
	*aac(6′)-It*	441	AF031328	1… 441	*Acinetobacter*
	*aac(6′)-Iu*	441	AF031329	1… 441	*Acinetobacter*
	*aac(6′)-Iv*	441	AF031330	1… 441	*Acinetobacter*
	*aac(6′)-Iw*	441	AF031331	1… 441	*Acinetobacter*
	*aac(6′)-Ix*	441	AF031332	1… 441	*Acinetobacter*
	*aac(6′)-Iy*	438	AF144880	3452… 3979	*Salmonella*
	*aac(6′)-Iz*	462	AF140221	390… 851	*Stenotrophomonas*
	*aac(6′)-Iaa*	438	NC_003197	1707358… 1707795	*Salmonella*
	*aac(6′)-Iad*	435	AB119105	1… 435	*Acinetobacter*
	*aac(6′)-Iae*	552	AB104852	1935… 2486	*Pseudomonas*, *Salmonella*
	*aac(6′)-Iaf*	552	AB462903	1200… 1751	*Pseudomonas*
	*aac(6′)-Iai*	567	EU886977	544… 1110	*Pseudomonas*
	*aac(6′)-I30*	555	AY289608	1524… 2078	*Salmonella*
	*aac(6′)-31*	519	AJ640197	2474… 2992	*Acinetobacter*
	*aac(6′)-32*	555	EF614235	2247… 2801	*Pseudomonas*
	*aac(6′)-33*	555	GQ337064	1203… 1757	*Pseudomonas*
	*aac(6′)-IIa*	555	M29695	707… 1261	*Aeromonas*, *Klebsiella*, *Pseudomonas*, *Salmonella*
	*aac(6′)-IIb*	543	L06163	532… 1074	*Pseudomonas*
	*aac(6′)-IIc*	582	AF162771	62… 643	*Enterobacter*, *Klebsiella*, *Pseudomonas*
	*aac(6′)-Iid;*				
	*see ant(3″)-Ih- aac(6′)-IId*				
	*aac(6′)-III;*			
	*see aac(6′)-Ic*			
	*aac(6′)-IV*	435	X55353	279… 713	*Enterobacter*
	*aacA29*	381	AY139599	768… 1148	Unknown
	*aacA43*	564	HQ247816	639… 1202	*Klebsiella*
	*apmA*	822	FN806789	2858… 3682	*Staphylococcus*
	*sat2*^a^	525	X51546	518… 1042	*Acinetobacter, Enterobacter*, *Escherichia*, *Klebsiella*, *Proteus*, *Pseudomonas*,* Salmonella*, *Shigella*, *Vibrio*
	*sat3*^a^	543	Z48231	221… 763	*Escherichia*
	*sat4*^a^	543	X92945	38870… 39412	*Campylobacter*, *Enterococcus*, *Staphylococcus*,*Streptococcus*
	*aac(6′)*-*aph(2″)*	1440	M13771	304… 1743	*Enterococcus*, *Lactobacillus*, *Staphylococcus*,*Streptococcus*
ACT–PHT	*aph(2″-Ia; see*				
	*aac(6′)-aph(2″)*				
MET	*armA*	774	AY220558	1978… 2751	*Acinetobacter, Citrobacter*, *Enterobacter*, *Escherichia*, *Klebsiella*, *Salmonella*, *Serratia*
	*npmA*	660	AB261016	3069… 3728	*Escherichia*
	*rmtA*	756	AB120321	6677… 7432	*Pseudomonas*
	*rmtB*	756	AB103506	1410… 2165	*Enterobacter*, *Escherichia*, *Klebsiella*, *Pseudomonas*,* Serratia*
	*rmtC*	846	AB194779	6903… 7748	*Proteus*, *Salmonella*
	*rmtD*	744	DQ914960	8889… 9632	*Klebsiella*, *Pseudomonas*
	*rmtD2*	744	HQ401565	14139… 14882	*Citrobacter*, *Enterobacter*
	*rmtE*	822	GU201947	55… 876	*Escherichia*
NUT	*aadA1*	972	X02340	223… 1194	*Acinetobacter, Aeromonas*, *Enterobacter*, *Escherichia*, *Klebsiella*, *Proteus*, *Pseudomonas*, *Salmonella*, *Shigella*, *Vibrio*
	*aadA1b*	792	M95287	3320… 4111	*Pseudomonas*, *Serratia*
	*aadA2*	780	X68227	166… 945	*Acinetobacter, Aeromonas*, *Citrobacter*, *Enterobacter*, *Escherichia*, *Klebsiella*, *Proteus*, *Pseudomonas*, *Salmonella*, *Shigella*, *Staphylococcus*, *Vibrio*, *Yersinia*
	*aadA3*	792	AF047479	1296… 2087	*Escherichia*
	*aadA4*	789	Z50802	1306… 2094	*Acinetobacter, Aeromonas*,* Escherichia*,* Pseudomonas*,
	*aadA5*	789	AF137361	64… 852	*Acinetobacter, Aeromonas*, *Escherichia*, *Pseudomonas*, *Salmonella*, *Shigella*, *Staphylococcus*, *Vibrio*
	*aadA6*	846	AF140629	61… 906	*Pseudomonas*
	*aadA7*	798	AF224733	32… 829	*Escherichia*,* Salmonella*,* Vibrio*
	*aadA8*	792	AF326210	1… 792	*Klebsiella*,* Vibrio*
	*aadA8b*	792	AM040708	1174… 1965	*Escherichia*
	*aadA9*	837	AJ420072	26773… 27609	*Corynebacterium*
	*aadA10*	834	U37105	2807… 3640	*Pseudomonas*
	*aadA11*	846	AY144590	1… 846	*Pseudomonas*, *Riemerella*
	*aadA12*	792	AY665771	1… 792	*Escherichia*,* Salmonella*,* Yersinia*
	*aadA13*	798	AY713504	1… 798	*Escherichia*,* Pseudomonas*,* Yersinia*
	*aadA14*	786	AJ884726	540… 1325	*Pasteurella*
	*aadA15*	792	DQ393783	1800… 2591	*Pseudomonas*
	*aadA16*	846	EU675686	3197… 4042	*Escherichia*,* Klebsiella*,* Vibrio*
	*aadA17*	792	FJ460181	774… 1565	*Aeromonas*
	*aadA21*	792	AY171244	47… 838	*Salmonella*
	*aadA22*	792	AM261837	74… 865	*Escherichia*,* Salmonella*
	*aadA23*	780	AJ809407	119… 898	*Salmonella*
	*aadA24*	780	AM711129	1264… 2043	*Escherichia*,* Salmonella*
	*aadC*	477	V01282	225… 701	*Staphylococcus*
	*aadD aadE*; see *ant(6)-Ia*	771	AF181950	3176… 3946	*Staphylococcus*
	*ant(2″)-Ia*	543	X04555	1296… 1829	*Acinetobacter, Enterobacter*, *Escherichia*, *Klebsiella*, *Proteus*, *Pseudomonas*,* Salmonella*, *Serratia*,* Shigella*, *Vibrio*
	*ant(4′)-Ib*	771	AJ506108	209… 979	*Bacillus*
	*ant(4′)-IIa*	759	M98270	145… 903	*Pseudomonas*
	*ant(4′)-IIb*	756	AY114142	1061… 1816	*Pseudomonas*
	*ant(6)-Ia*	909	AF330699	22… 930	*Enterococcus*,* Staphylococcus*
	*ant(6)-Ib*	858	FN594949	27482… 28339	*Campylobacter*
	*ant(9)-Ia*	783	X02588	331… 1113	*Enterococcus*,* Staphylococcus*
	*ant(9)-Ib*	768	M69221	271… 1038	*Enterococcus*,* Staphylococcus*
	*spc*; see *ant(9)-Ia*				
	*sph*	801	X64335	6557… 7354	*Escherichia*, *Pseudomonas*,* Salmonella*
	*str*	849	X92946	18060… 18908	*Enterococcus*, *Staphylococcus, Lactococcus*
NUT–ACT	*ant(3″)-Ih-aac(6′)-IId*	1392	AF453998	3555… 4946	*Serratia*
PHT	*aph(2″)-Ib*	900	AF337947	272… 1171	*Enterococcus*, *Escherichia*
	*aph(2″)-Ic*	921	U51479	196… 1116	*Enterococcus*
	*aph(2″)-Id*	906	AF016483	131… 1036	*Enterococcus*
	*aph(2″)-Ie*	906	AY743255	131… 1036	*Enterococcus*
	*aph(3′)-Ia*	816	J01839	1162… 1977	*Escherichia*, *Klebsiella*, *Pseudomonas*,* Salmonella*
	*aph(3′)-Ib*	816	M20305	779… 1594	*Escherichia*
	*aph(3′)-Ic*	816	X625115	410… 1225	*Acinetobacter*, *Citrobacter*, *Escherichia*, *Klebsiella*, *Salmonella*, *Serratia*, *Yersinia*
	*aph(3′)-Id*	816	Z48231	820… 1635	*Escherichia*
	*aph(3′)-IIa*	795	X57709	1… 795	*Escherichia*,* Pseudomonas*,* Salmonella*
	*aph(3′)-IIb*	807	X90856	388… 1194	*Pseudomonas*
	*aph(3′)-IIc*	813	AM743169	2377498… 2378310	*Stenotrophomonas*
	*aph(3′)-III*	795	M26832	604… 1398	*Bacillus*, *Campylobacter*, *Enterococcus*, *Staphylococcus*, *Streptococcus*
	*aph(3′)-IV*	789	X03364	277… 1065	*Bacillus*
	*aph(3′)-Va*	807	K00432	307… 1113	*Streptomyces*
	*aph(3′)-Vb*	792	M22126	373… 1164	*Streptomyces*
	*aph(3′)-Vc*	795	S81599	282… 1076	*Micromonospora*
	*aph(3′)-Va*	780	X07753	103… 882	*Acinetobacter*, *Pseudomonas*
	*aph(3′)-VIb*	780	AJ627643	4934… 5713	*Alcaligenes*
	*aph(3′)-VIIa*	753	M29953	131… 1036	*Campylobacter*
	*aph(3′)-VIII*	804	AF182845	1… 804	*Streptomyces*
	*aph(3′)-XV*	795	Y18050	4758… 5552	*Achromobacter*, *Citrobacter*, *Pseudomonas*
	*aph(3″)-Ia*	819	M16482	501… 1319	*Streptomyces*
	*aph(3″)-Ib*	801	AB366441	11310… 12110	*Enterobacter*, *Escherichia*, *Klebsiella*, *Pasteurella*, *Pseudomonas*,* Salmonella*, *Shigella*, *Yersinia*, *Vibrio*
	*aph(4)-Ia*	1026	V01499	231… 1256	*Escherichia*
	*aph(4)-Ib*	999	X03615	232… 1230	*Streptomyces*
	*aph(6)-Ia*	924	AY971801	1… 924	*Streptomyces*
	*aph(6)-Ib*	924	X05648	382… 1305	*Streptomyces*
	*aph(6)-Ic*	801	X01702	485… 1285	*Escherichia*,* Pseudomonas*,* Salmonella*
	*aph(6)-Id*	837	M28829	866… 1702	*Enterobacter*, *Escherichia*, *Klebsiella*, *Pasteurella*, *Pseudomonas*,* Salmonella*, *Shigella*, *Yersinia*, *Vibrio*
	*aph(7″)-Ia*	999	X03615	232… 1230	*Streptomyces*
	*aph(9)-Ia*	996	U94857	151… 1146	*Legionella*
	*aph(9)-Ib*	993	U70376	7526… 8518	*Streptomyces*

* Last update: January 6th 2012. This table was adapted from Elbourne and Hall (2006), Magnet and Blanchard (2005), Partridge et al. (2009), Ramirez and Tolmansky (2010), Shaw et al. (1993), Vakulenko and Mobashery (2003), and data provided by B. Guerra, B. Aranda, D. Avsaroglu, B. Ruiz del Castillo, and R. Helmuth, on behalf of the Med-Vet Net (EU Network of Excellence) WP29 Project Group. The data were collected within the subproject “AME's,” with following participants representing their institutions: Agnes Perry Guyomard (ANSES), Dik Mevius (CVI), Yvonne Agerso (DTU), Katie Hopkins (HPA), Silvia Herrera (ISCIII), Alessandra Carattoli (ISS), Antonio Battisti (IZS-Rome), Stefano Lollai (IZS-Sardegna,), Lotte Jacobsen (SSI), Béla Nagy (VMRI), M. Rosario Rodicio and M. C. Mendoza (University of Oviedo, UO), Luis Martínez-Martínez (University Hospital of Valdecilla, HUV), and Bruno Gonzalez-Zorn (UCM).

ACT: Acetyltransferase; MET: Methyltransferase; NUT: Nucleotidyltransferase; PHT: Phosphotransferase.

a Although the sat genes are not aminoglycoside resistance determinants, they encode streptothricin-acetyltransferases, for convenience they are included in this table.

**Table 2:** **β-lactamases and ESBLs families**.

**Amber class A β-lactamases and ESBLs**	Number of variants^*^	**Amber class B β-lactamases and MBLs**	Number of variants^*^	**Amber class C β-lactamases and ESBLs**	Number of variants^*^	**Amber class D β-lactamases and ESBLs**	Number of variants^*^
*bla*_ACI_	1	*bla*_B_	13	*bla*_ACC_^a^	5	*ampH*	1
*bla*_AER_	1	*bla*_CGB_	2	*bla*_ACT_^a^	14	*ampS*	1
*bla*_AST_	1	*bla*_DIM_	1	*bla*_ADC_	54	*bla*_LCR_	1
*bla*_BEL_	3	*bla*_EBR_	1	*bla*_BIL_	1	*bla*_NPS_	1
*bla*_BES_	1	*bla*_GIM_	1	*bla*_BUT_	2	*bla*_OXA_^a^	247
*bla*_BIC_	1	*bla*_GOB_	18	*bla*_CFE_^a^	1	*loxA*	1
*bla*_BPS_	5	*bla*_IMP_^a^	37	*bla*_CMG_	1		
*bla*_CARB_	14	*bla*_IND_^a^	7	*bla*_CMY_^a^	92		
*bla*_CIA_	1	*bla*_JOHN_	1	*bla*_DHA_^a^	8		
*bla*_CGA_	1	*bla*_MUS_	1	*bla*_FOX_^a^	10		
*bla*_CKO_	5	*bla*_NDM_	6	*bla*_LAT_^a^	1		
*bla*_CME_	2	*bla*_SIM_	1	*bla*_LEN_^c^	26		
*bla*_CTX-M_^a^	130	*bla*_SPM_	1	*bla*_MIR_^a^	5		
*bla*_DES_	1	*bla*_TUS_	1	*bla*_MOR_	1		
*bla*_ERP_	1	*bla*_VIM_^a^	34	*bla*_MOX_^a^	8		
*bla*_FAR_	1	*cepA*	7	*bla*_OCH_	7		
*bla*_FONA_	6	*cfiA*	16	*bla*_OKP-A_^c^	16		
*bla*_GES_^a^^,^^b^	22	*cphA*	8	*bla*_OKP-B_^c^	20		
*bla*_HERA_	8	*imiH*	1	*bla*_OXY_^c^	23		
*bla*_IMI_	3	*imiS*	1	*bla*_TRU_	1		
*bla*_KLUA_^d^	12			*bla*_ZEG_	1		
*bla*_KLUC_^d^	2			*cepH*	1		
*bla*_KLUG_	1						
*bla*_KLUY_	4						
*bla*_KPC_^a^	12						
*bla*_LUT_	6						
*bla*_MAL_	2						
*bla*_MOR_	1						
*bla*_NMC-A_	1						
*bla*_PER_^a^	7						
*bla*_PME_	1						
*bla*_PSE_	4						
*bla*_RAHN_	2						
*bla*_ROB_	1						
*bla*_SED_	1						
*bla*_SFC_	1						
*bla*_SFO_	1						
*bla*_SHV_^a^	166						
*bla*_SME_^a^	3						
*bla*_TEM_^a^	201						
*bla*_TLA_	1						
*bla*_TOHO_	1						
*bla*_VEB_^a^	7						
*bla*_Z_	1						
*cdiA*	1						
*cfxA*	6						
*cumA*	1						
*hugA*	1						
*penA*	1						

* Last update: June 8th 2012.

a According to http://www.lahey.org/Studies.

b GES and IBC-type ESBLs have all been renamed as *bla*_GES_ according to Weldhagen et al. (2006).

c According to http://www.pasteur.fr/ip/easysite/go/03b-00002u-03q/beta-lactamase-enzyme-variants.

d bla_KLUA_, bla_KLUC_, bla_KLUG_, and bla_KLUY_ seem to be the chromosomal progenitors of acquired CTX-M group 2, 1, 8, and 9 genes, respectively (Saladin et al., 2002; Olson et al., 2005).

**Table 3:** Acquired phenicol resistance genes^*^.

**Mechanism**	**Group**	**Gene**	**Gene(s) included**	**Length (nt)**	**Accession number**	**Coding region**	**Genera**
Efflux	Type E-1	*cmlA1*	*cmlA, cmlA2, cmlA4, cmlA5, cmlA6, cmlA7, cmlA8, cmlA10, cmlB*	1260	M64556	601… 1860	*Acinetobacter*, *Aeromonas*, *Arcanobacterium*, *Enterobacter*, *Escherichia*, *Klebsiella*, *Laribacter*, *Pseudomonas*, *Salmonella*, *Serratia*, *Staphylococcus*
	Type E-2	*cml*	*−*	903	M22614	427… 1335	*Escherichia*
	Type E-3	*floR*	*cmlA*-like, *flo*, *pp-flo, cmlA9*	1215	AF071555	4445… 5659	*Acinetobacter*, *Aeromonas*, *Bordetella*, *Escherichia*, *Pasteurella*, *Salmonella*, *Stenotrophomonas*, *Vibrio*
	Type E-4	*fexA*	*−*	1428	AJ549214	177… 1604	*Bacillus*, *Staphylococcus*
	Type E-5	*cml*	*−*	1179	X59968	508… 1686	*Streptomyces*
	Type E-6	*cmlv*	*−*	1311	U09991	28… 1338	*Streptomyces*
	Type E-7	*cmrA*	*cmr*	1176	Z12001	993… 2168	*Rhodococcus*
	Type E-8	*cmr*	*cmx*	1176	U85507	3518… 4693	*Corynebacterium*
	*−*	*cmlB1*	*−*	1266	AM296481	776… 2041	*Bordetella*
	*−*	*fexB*	*−*	1410	JN192453	10637… 12046	*Enterococcus*
	*−*	*pexA*	*−*	1248	HM537013	24055… 25302	Uncultured
Inactivating enzyme	Type A-1	*catA1*	*cat*, *catI*, *pp-cat*	660	V00622	244… 903	*Acinetobacter*, *Corynebacterium*, *Escherichia*, *Klebsiella*, *Salmonella*, *Shigella*
	Type A-2	*catA2*	*cat*, *catII*	642	X53796	187… 828	*Aeromonas, Agrobacterium*, *Escherichia*, *Haemophilus*, *Legionella*, *Klebsiella*, *Photobacterium*, *Salmonella*, *Vibrio*
	Type A-3	*catA3*	*cat*, *catIII*	642	X07848	272… 913	*Actinobacillus*, *Edwardsiella*, *Klebsiella*, *Mannheimia*, *Pasteurella*, *Shigella*
	Type A-4	*cat*	*−*	654	M11587	880… 1533	*Proteus*
	Type A-5	*cat*	*−*	663	P20074^$^	1002758… 1003420	*Streptomyces*
	Type A-6	*cat86*	*−*	663	K00544	145… 807	*Bacillus*
	Type A-7	*cat(pC221)*	*cat*, *catC*	648	X02529	2267… 2914	*Bacillus*, *Enterococcus*, *Lactobacillus*, *Staphylococcus*, *Streptococcus*
	Type A-8	*cat(pC223)*	*cat*	648	AY355285	1000… 1647	*Enterococcus*, *Lactococcus*, *Listeria*, *Staphylococcus*
	Type A-9	*cat(pC194)*	*cat*, *cat-TC*	651	NC_002013	1260… 1910	*Bacillus*, *Enterococcus*, *Lactobacillus*, *Staphylococcus*, *Streptococcus*
	Type A-10	*cat*	*−*	687	AY238971	1055… 1741	*Bacillus*
	Type A-11	*catP*	*catD*	624	U15027	2953… 3576	*Clostridium*, *Neisseria*
	Type A-12	*catS*	*−*	492^§^	X74948	1… 492	*Streptococcus*
	Type A-13	*cat*	*−*	624	M35190	309… 932	*Campylobacter*
	Type A-14	*cat*	*−*	651	S48276	479… 1129	*Listonella*, *Photobacterium*, *Proteus*, *Vibrio*
	Type A-15	*catB*	*−*	660	M93113	145… 804	*Clostridium*
	Type A-16	*catQ*	*−*	660	M55620	459… 1118	*Clostridium*
	Type B-1	*catB1*	*cat*	630	M58472	148… 777	*Agrobacterium*
	Type B-2	*catB2*	*−*	633	AF047479	5957… 6589	*Acinetobacter*, *Aeromonas*, *Bordetella*, *Escherichia*, *Klebsiella*, *Pasteurella*, *Pseudomonas*, *Salmonella*
	Type B-3	*catB3*	*catB4*, *catB5*, *catB6*, *catB8*	633	AJ009818	883… 1515	*Acinetobacter*, *Aeromonas*, *Bordetella*, *Comamonas*, *Enterobacter*, *Escherichia*, *Klebsiella*, *Kluyvera*, *Morganella*, *Proteus*, *Pseudomonas*, *Salmonella*, *Serratia*, *Stenotrophomonas*
	Type B-4	*catB7*	*−*	639	AF036933	177… 815	*Pseudomonas*
	Type B-5	*catB9*	*−*	630	AF462019	27… 656	*Vibrio*
	Type B-6	*catB10*	*−*	633	AJ878850	1197… 1829	*Pseudomonas*
rRNA methylase	*−*	*cfr*^$^	*−*	1050	AJ579365	6290… 7339	*Bacillus*, *Enterococcus*, *Escherichia*, *Jeotgalicoccus*, *Macrococcus*, *Proteus*, *Staphylococcus*

* Last update: December 16th 2011. Adapted from Partridge et al. (2009), Roberts and Schwarz (2009), Schwarz et al. (2004), and nucleotide BLAST searches.

§ Partial sequence.

$ The multidrug resistance gene cfr confers resistance against phenicols, lincosamides, oxazolidinones, pleuromutilins, and streptogramin A (see Table 4; Kehrenberg et al., 2007).

To the subsection dealing with the “*Resistance mechanisms”* of the AMINOGLYCOSIDES we would like to add that to date six additional methylases have been reported, i.e., *npmA*, *rmt*A, *rmtB*, *rmtC*, *rmtD*, and *rmtE* (Courvalin, 2008; Doi et al., 2008; Davis et al., 2010). Futhermore, that within the three major classes (AAC, ANT, and APH) an additional subdivision can be made based on the enzymes' target sites within the aminoglycoside molecules: i.e., there are four acetyltransferases: AAC(1), AAC(2′), AAC(3), and AAC(6′); five nucleotidyltransferases: ANT(2″), ANT(3″), ANT(4′), ANT(6), and ANT(9); and seven phosphotransferases: APH(2″), APH(3′), APH(3″), APH(4), APH(6), APH(7″), and APH(9).

To the subsection β-LACTAM, *Resistance, mechanisms* we would like to add that in recent years acquired genes encoding ESBLs have become a major concern (Bradford, 2001). Over time, the genes for the parent enzymes *bla*_TEM−1_, *bla*_TEM−2_, and *bla*_SHV−1_ have undergone point mutations which resulted in amino acid substitutions that changed the substrate spectrum to that of ESBLs, starting with *bla*_TEM−3_ and *bla*_SHV−2_ (Bradford, 2001).

Because chloramphenicol is not an actual antibiotic class the subsection of CHLORAMPHENICOL should be called PHENICOLS. Concerning the history of PHENICOLS, it is worthwhile to know the first antibiotic, chloramphenicol, originally referred to as chloromycetin, was isolated already in 1947 from *Streptomyces venezuelae* (Ehrlich et al., 1947).

Besides the inactivating enzymes (chloramphenicol acetyltransferases), there are also reports on other phenicol resistance systems, such as the inactivation by phosphotransferases, mutations of the target site, permeability barriers, and efflux systems (Schwarz et al., 2004). Of the latter mechanism, *cmlA* and *floR* are the most commonly known genes in Gram-negative bacteria (Bissonnette et al., 1991; Briggs and Fratamico, 1999).

The macrolides (subsection MACROLIDES–LINCOSAMIDES–STREPTOGRAMIN B) have a similar mode of antibacterial action, comparable antibacterial spectra and in part overlapping binding sites at the ribosome as two other antibiotic classes, i.e., lincosamides and streptogramin antibiotics (comprising streptogramin A and B compounds that act synergistically). Consequently, these antibiotics, although chemically distinct, have been clustered together as MLS antibiotics (Roberts, 1996). Macrolides, lincosamides and streptogramins all inhibit protein synthesis by binding to the 50S ribosomal subunit of bacteria (Weisblum, 1995; Roberts, 2002).

To *Resistance mechanisms* of the subsection MACROLIDES–LINCOSAMIDES–STREPTOGRAMIN B. Shortly after the introduction of erythromycin into clinical setting in the 1950s, bacterial resistance to this antibiotic was reported for the first time in staphylococci (Weisblum, 1995). Since then a large number of bacteria have been identified that are resistant to MLS due to the presence of various different genes. The resistance determinants responsible include rRNA methylases that modify the ribosomal target sites, ABC transporters, and efflux proteins of the Major Facilitator Superfamily, as well as genes for inactivating enzymes (Roberts et al., 1999; Roberts, 2008). The latter group can be further subdivided into esterases, lyases, phosphorylases, and transferases (Table 4).

**Table 4:** Acquired macrolide-lincosamide-streptogramin B (MLS) resistance genes^*^.

**Mechanism**	**Gene**	**Gene(s) included**	**Length (nt)**	**Accession number**	**Coding region**	**Genera**
Efflux	*car*(A)	−	1656	M80346	411… 2066	*Streptomyces*
	*lmr*(A)	−	1446	X59926	318… 1763	*Streptomyces*
	*lsa*(A)	*abc-23*	1497	AY225127	41… 1537	*Enterococcus*
	*lsa*(B)	*orf3*	1479	AJ579365	4150… 5628	*Staphylococcus*
	*lsa*(C)	−	1479	HM990671	5193… 6671	*Gardnerella*, *Streptococcus*
	*lsa*(E)	−	1485	JQ861959	6673… 8157	*Enterococcus*, *Staphylococcus*
	*mef*(A)	*mef*(E)	1218	U70055	314… 1531	*Acinetobacter*, *Bacteroides*, *Citrobacter*, *Clostridium*, *Corynebacterium*, *Enterococcus*, *Enterobacter*, *Escherichia*, *Fusobacterium*, *Gemella*, *Haemophilus, Klebsiella*, *Lactobacillus*, *Micrococcus*, *Morganella*, *Neisseria*, *Pantoea*, *Providencia*, *Proteus*, *Ralstonia*, *Rothia, Pseudomonas*, *Salmonella*, *Serratia*, *Staphylococcus*, *Streptococcus*, *Stenotrophomonas, Ureaplasma*
	*mef*(B)	−	1230	FJ196385	11084… 12313	*Escherichia*
	*msr*(A)	*msr*(B), *msr*(SA)	1467	X52085	343… 1809	*Corynebacterium*, *Enterobacter*, *Enterococcus*, *Gemella*, *Pseudomonas*, *Staphylococcus*, *Streptococcus, Ureaplasma*
	*msr*(C)	−	1479	AY004350	496… 1974	*Enterococcus*
	*msr*(D)	*mel, orf5*	1464	AF274302	2462… 3925	*Acinetobacter*, *Bacteroides*, *Citrobacter*, *Clostridium*, *Corynebacterium*, *Enterococcus*, *Enterobacter*, *Escherichia*, *Gemella*, *Fusobacterium*, *Klebsiella*, *Morganella*, *Neisseria*, *Proteus*, *Providencia*, *Pseudomonas*, *Ralstonia*, *Staphylococcus*, *Streptococcus*, *Serratia*, *Stenotrophomonas, Ureaplasma*
	*msr*(E)	*mel*	1476	AY522431	20650… 22125	*Acinetobacter, Citrobacter, Escherichia, Klebsiella, Mannheimia, Pasteurella, Serratia*
	*ole*(B)	−	1710	L36601	1421… 3130	*Streptomyces*
	*ole*(C)	−	978	L06249	1528… 2505	*Streptomyces*
	*srm*(B)	−	1653	X63451	558… 2210	*Streptomyces*
	*tlc*(C)	−	1647	M57437	277… 1923	*Streptomyces*
	*vga*(A)	*vga*	1569	M90056	909… 2477	*Staphylococcus*
	*vga*(A)_LC_	*vga*	1569	DQ823382	1… 1569	*Staphylococcus*
	*vga*(B)	−	1659	U82085	629… 2287	*Enterococcus*, *Staphylococcus*
	*vga*(C)	−	1569	NC_013034	12570… 14138	*Staphylococcus*
	*vga*(D)	−	1578	GQ205627	1394… 2971	*Enterococcus*
	*vga*(E)	−	1575	FR772051	8741… 10315	*Staphylococcus*
Inactivating enzyme^a^	*ere*(A)	−	1221	AY183453	2730… 3950	*Achromobacter, Aermonas, Citrobacter*, *Enterobacter*, *Escherichia, Klebsiella, Laribacter, Pantoeae, Providencia, Pseudomonas, Serratia, Salmonella, Staphylococcus, Stenotrophomonas*
	*ere*(B)	−	1260	X03988	383… 1642	*Acinetobacter*, *Citrobacter*, *Enterobacter*, *Escherichia*, *Klebsiella*, *Proteus*, *Pseudomonas*, *Staphylococcus*
Inactivating enzyme^b^	*vgb*(A)	*vgb*	900	M20129	641… 1540	*Enterococcus*, *Staphylococcus*
	*vgb*(B)	−	888	AF015628	399… 1286	*Staphylococcus*
Inactivating enzyme^c^	*lnu*(A)	*lin*(A), *lin*(A')	486	M14039	413… 898	*Clostridium*, *Lactobacillus*, *Staphylococcus*
	*lnu*(B)	*lin*(B)	804	AJ238249	127… 930	*Clostridium*, *Enterococcus*, *Staphylococcus*, *Streptococcus*
	*lnu*(C)	−	495	AY928180	1150… 1644	*Haemophilus, Streptococcus*
	*lnu*(D)	−	495	EF452177	19… 513	*Streptococcus*
	*lnu*(F)	*lin*(F), *lin*(G), *linF*	822	EU118119	1030… 1851	*Escherichia, Salmonella*
	*vat*(A)	−	660	L07778	258… 917	*Staphylococcus*
	*vat*(B)	−	639	U19459	67… 705	*Enterococcus*, *Staphylococcus*
	*vat*(C)	−	639	AF015628	1307… 1945	*Staphylococcus*
	*vat*(D)	*sat*(A)	630	L12033	162… 791	*Enterococcus*
	*vat*(E)	*sat*(G), *vat* (E-3)–*vat(E-8)*	645	AF139725	63… 707	*Enterococcus*, *Lactobacillus*
	*vat*(F)	−	666	AF170730	70… 735	*Yersinia*
	*vat*(H)	*vat*(G)	651	GQ205627	3037… 3687	*Enterococcus*
Inactivating enzyme^d^	*mph*(A)	*mph*(K)	906	D16251	1626… 2531	*Aeromonas*, *Escherichia*, *Citrobacter*, *Enterobacter*, *Klebsiella*, *Pantoeae*, *Pseudomonas*, *Proteus*, *Serratia*, *Shigella*, *Stenotrophomonas*
	*mph*(B)	*mph*(B)	909	D85892	1159… 2067	*Escherichia*, *Enterobacter*, *Proteus*, *Pseudomonas*
	*mph*(C)	*mph*(BM)	900	AF167161	5665… 6564	*Staphylococcus*, *Stenotrophomonas*
	*mph*(D)	−	840^§^	AB048591	1… 840	*Escherichia*, *Klebsiella*, *Pantoea*, *Proteus*, *Pseudomonas*, *Stenotrophomonas*
	*mph*(E)	*mph*, *mph1*, *mph2*	885	DQ839391	12873… 13757	*Acinetobacter, Citrobacter, Escherichia, Klebsiella, Mannheimia, Pasteurella, Serratia*
	*mph*(F)	*mph*(B), *mph*(E)	900	AM206957	4187… 5086	Unknown
rRNA methylase	*cfr*^$^	*−*	1050	AM408573	10028… 11077	*Bacillus*, *Enterococcus*, *Escherichia*, *Jeotgalicoccus*, *Macrococcus*, *Proteus*, *Staphylococcus*
	*erm*(A)	*erm*(TR)	732	X03216	4551… 5282	*Aggregatibacter*, *Bacteriodes*, *Enterococcus*, *Helcococcus*, *Peptostreptococcus*, *Prevotella*, *Staphylococcus*, *Streptococcus*
	*erm*(B)	*erm*(2), *erm*(AM), *erm*(AMR), *erm*(BC), *erm*(BP), *erm*(Z), *erm*(BZ1, BZ2), *erm*(IP), *erm*(P), *erm, erm*(80)	738	M36722	714… 1451	*Aggregatibacter*, *Acinetobacter*, *Aerococcus*, *Arcanobacterium*, *Bacillus*, *Bacteriodes*, *Citrobacter*, *Corynebacterium*, *Clostridium*, *Enterobacter*, *Escherichia*, *Eubacterium*, *Enterococcus*, *Fusobacterium*, *Gemella*, *Haemophilus*, *Klebsiella*, *Lactobacillus*, *Micrococcus*, *Neisseria*, *Pantoeae*, *Pediococcus*, *Peptostreptococcus*, *Porphyromonas*, *Proteus*, *Pseudomonas*, *Ruminococcus*, *Rothia*, *Serratia*, *Staphylococcus*, *Streptococcus*, *Ureaplasma*, *Treponema*, *Wolinella*
	*erm*(C)	*erm*(IM), *erm*(M)	735	M19652	988… 1722	*Aggregatibacter*, *Actinomyces*, *Arcanobacterium, Bacillus*, *Bacteriodes*, *Clostridium, Corynebacterium*, *Escherichia, Eubacterium*, *Enterococcus*, *Haemophilus*, *Lactobacillus*, *Macrococcus, Micrococcus*, *Neisseria*, *Prevotella*, *Peptostreptococcus*, *Staphylococcus*, *Streptococcus*, *Wolinella*
	*erm*(D)	*erm*(J), *erm*(K)	864	M29832	430… 1293	*Bacillus*, *Salmonella*
	*erm*(E)	*erm*(E2)	1146	X51891	190… 1335	*Bacteroides*, *Eubacterium*, *Fusobacterium*, *Ruminococcus*, *Saccharopolyspora, Shigella*, *Streptomyces*
	*erm*(F)	*erm*(FS), *erm*(FU)	801	M14730	241… 1041	*Aggregatibacter*, *Actinomyces*, *Bacteroides*, *Capnocytophaga, Clostridium*, *Corynebacterium*, *Eubacterium*, *Enterococcus*, *Fusobacterium*, *Gardnerella*, *Haemophilus*, *Lactobacillus*, *Mobiluncus*, *Neisseria*, *Porphyromonas*, *Prevotella*, *Peptostreptococcus*, *Ruminococcus*, *Shigella*, *Selenomonas*, *Staphylococcus*, *Streptococcus*, *Treponema*, *Veillonella*, *Wolinella*
	*erm*(G)	−	735	M15332	672… 1406	*Bacillus*, *Bacteroides*, *Catenibacterium*, *Lactobacillus*, *Prevotella*, *Porphyromonas*, *Staphylococcus*
	*erm*(H)	*car*(B)	900	M16503	244… 1143	*Streptomyces*
	*erm*(I)	*mdm*(A)	−	−	−	*Streptomyces*
	*erm*(N)	*tlr*(D)	876	X97721	160… 1035	*Streptomyces*
	*erm*(O)	*lrm*, *srm*(A)	783	M74717	40… 822	*Streptomyces*
	*erm*(Q)	−	774	L22689	262… 1035	*Aggregatibacter*, *Bacteroides*, *Clostridium*, *Staphylococcus*, *Streptococcus*, *Wolinella*
	*erm*(R)	−	1023	M11276	333… 1355	*Aeromicrobium, Arthrobacter*
	*erm*(S)	*erm*(SF), *tlr*(D)	960	M19269	460… 1419	*Streptomyces*
	*erm*(T)	*erm*(GT), *erm*(LF)	735	M64090	168… 902	*Enterococcus*, *Lactobacillus*, *Staphylococcus, Streptococcus*
	*erm*(U)	*lrm*(B)	837	X62867	361… 1197	*Streptomyces*
	*erm*(V)	*erm*(SV)	780	U59450	397… 1176	*Eubacterium*, *Fusobacterium*, *Streptomyces*
	*erm*(W)	*myr*(B)	936	D14532	1039… 1974	*Micromonospora*
	*erm*(X)	*erm*(CD), *erm*(Y)	855	M36726	296… 1150	*Arcanobacterium*, *Bifidobacterium*, *Corynebacterium*, *Propionibacterium*
	*erm*(Y)	*erm*(GM)	735	AB014481	556… 1290	*Staphylococcus*
	*erm*(Z)	*srm*(D)	849	AM709783	2817… 3665	*Streptomyces*
	*erm*(30)	*pikR1*	1011	AF079138	1283… 2293	*Streptomyces*
	*erm*(31)	*pikR2*	969	AF079138	154… 1122	*Streptomyces*
	*erm*(32)	*tlr*(B)	843	AJ009971	1790… 2632	*Streptomyces*
	*erm*(33)	−	732	AJ313523	163… 894	*Staphylococcus*
	*erm*(34)	−	846	AY234334	355… 1200	*Bacillus*
	*erm*(35)	−	801	AF319779	33… 833	*Bacteriodes*
	*erm*(36)	−	846	AF462611	186… 1031	*Micrococcus*
	*erm*(37)	*erm*(MT)	540	AE000516	2229013… 2229552	*Mycobacterium*
	*erm*(38)	−	1161	AY154657	63… 1223	*Mycobacterium*
	*erm*(39)	−	741	AY487229	2153… 2893	*Mycobacterium*
	*erm*(40)	−	756	AY570506	2035… 2790	*Mycobacterium*
	*erm*(41)	−	522	EU590124	258… 779	*Mycobacterium*
	*erm*(42)	*erm*(MI)	906	FR734406	1… 906	*Mannheimia, Pasteurella, Photobacterium*

* Last update: January 6th 2012. Adapted from http://faculty.washington.edu/marilynr/

§ Partial sequence.

$ The multidrug resistance gene cfr confers resistance against phenicols, lincosamides, oxazolidinones, pleuromutilins, and streptogramin A (see Table 3; Kehrenberg et al., 2007).

a Esterase,

b Lyase,

c Transferase, and

d Phosphorylase.

**Table 5:** Acquired quinolone resistance genes^*^.

**Gene**	**Length (nt)**	**Accession number**	**Coding region**	**Genera**
*qepA*	1536	AB263754	7052… 8587	*Escherichia*
*qepA2*	1536	EU847537	1672… 3207	*Escherichia*
*qnrA1*^a^	657	AY070235	303… 959	*Citrobacter*, *Escherichia*, *Klebsiella*, *Proteus*
*qnrA2*^a^	657	AY675584	1… 657	*Klebsiella*, *Shewanella*
*qnrA3*^a^	657	DQ058661	1… 657	*Shewanella*
*qnrA4*^a^	657	DQ058662	1… 657	*Shewanella*
*qnrA5*^a^	657	DQ058663	1… 657	*Shewanella*
*qnrA6*^a^	657	DQ151889	1… 657	*Proteus*
*qnrA7*^a^	657	GQ463707	1… 657	*Shewanella*
*qnrB1*^a^	645	DQ351241	37… 681	*Klebsiella*
*qnrB2*^a^	645	DQ351242	1… 645	*Citrobacter*, *Enterobacter*, *Klebsiella*, *Salmonella*
*qnrB3*^a^	645	DQ303920	37… 681	*Escherichia*
*qnrB4*^a^	645	DQ303921	4… 648	*Citrobacter, Enterobacter*, *Escherichia*, *Klebsiella*
*qnrB5*^a^	645	DQ303919	37… 681	*Salmonella*
*qnrB6*^a^	645	EF520349	37… 681	*Enterobacter*, *Panthoea*
*qnrB7*^a^	645	EU043311	1… 645	*Enterobacter*, *Klebsiella*
*qnrB8*^a^	645	EU043312	1… 645	*Citrobacter*
*qnrB9*^a^	645	EF526508	1… 645	*Citrobacter*
*qnrB10*^a^	645	DQ631414	37… 681	*Citrobacter*, *Enterobacter*, *Klebsiella*
*qnrB11*^a^	645	EF653270	4… 648	*Citrobacter*
*qnrB12*^a^	645	AM774474	2435… 3079	*Citrobacter*
*qnrB13*^a^	645	EU273756	37… 681	*Citrobacter*
*qnrB14*^a^	645	EU273757	37… 681	*Citrobacter*
*qnrB15*^a^	645	EU302865	37… 681	*Citrobacter*
*qnrB16*^a^	645	EU136183	37… 681	*Citrobacter*
*qnrB17*^a^	645	AM919398	37… 681	*Citrobacter*
*qnrB18*^a^	645	AM919399	37… 681	*Citrobacter*
*qnrB19*^a^	645	EU432277	1… 645	*Escherichia*, *Klebsiella*, *Salmonella*
*qnrB20*^a^	645	AB379831	37… 681	*Escherichia*
*qnrB21*^a^	645	FJ611948	1… 645	*Escherichia*
*qnrB22*^a^	645	FJ981621	37… 681	*Citrobacter*
*qnrB23*^a^	645	FJ981622	37… 681	*Citrobacter*
*qnrB24*^a^	645	HM192542	37… 681	*Citrobacter*
*qnrB25*^a^	645	HQ172108	1… 645	*Citrobacter*
*qnrB26*^a^	645	HM439644	1… 645	*Citrobacter*
*qnrB27*^a^	645	HM439641	1… 645	*Citrobacter*
*qnrB28*^a^	645	HM439643	1… 645	*Citrobacter*
*qnrB29*^a^	645	HM439649	37… 681	*Citrobacter*
*qnrB30*^a^	645	HM439650	37… 681	*Citrobacter*
*qnrB31*^a^	645	HQ418999	1… 645	*Klebsiella*
*qnrB32*^a^	645	JN173054	37… 681	*Citrobacter*
*qnrB33*^a^	645	JN173055	36… 680	*Citrobacter*
*qnrB34*^a^	645	JN173056	39… 683	*Citrobacter*
*qnrB35*^a^	645	JN173057	2307… 2951	*Citrobacter*
*qnrB36*^a^	645	JN173058	37… 681	*Citrobacter*
*qnrB37*^a^	645	JN173059	36… 680	*Citrobacter*
*qnrB38*^a^	645	JN173060	2307… 2951	*Citrobacter*
*qnrB39*^a^	−	NZ_ABWL02000005	−	*−*
*qnrB40*^a^	645	JN166689	16… 660	*Citrobacter*
*qnrB41*^a^	645	JN166690	37… 681	*Citrobacter*
*qnrB42*^a^	645	JN680743	1… 645	*Klebsiella*
*qnrB43*^a^	644	JQ349152	37… 680	*Escherichia*
*qnrB44*^a^	644	JQ349153	37… 680	*Escherichia*
*qnrB45*^a^	644	JQ349152	37… 680	*Escherichia*
*qnrB46*^a^	644	JQ349154	37… 680	*Escherichia*
*qnrB47*^a^	644	JQ349155	37… 680	*Escherichia*
*qnrB48*^a^	645	JQ762640	37… 681	*Citrobacter*
*qnrB49*^a^	645	JQ582718	37… 681	*Citrobacter*
*qnrB50*–*qnrB51* not public yet				
*qnrB52*^a^	645	EF488762	1… 645	*Proteus*
*qnrB53*^a^	645	HQ704413	37… 681	*Klebsiella*
*qnrB54*–*qnrB59* not public yet				
*qnrC*^a^	666	EU917444	1717… 2382	*Proteus*
*qnrD*^a^	645	EU692908	1… 645	*Escherichia*, *Morganella*, *Proteus*, *Providencia*, *Salmonella*
*qnrS1*^a^	657	AB187515	9737… 10393	*Enterobacter*, *Escherichia*, *Klebsiella*, *Proteus*, *Salmonella*, *Shigella*
*qnrS2*^a^	657	DQ485530	1… 657	*Aeromonas*, *Salmonella*
*qnrS3*^a^	>656	EU077611	<1… 656	*Escherichia*
*qnrS4*^a^	657	FJ418153	1… 657	*Salmonella*
*qnrS5*^a^	657	HQ631377	1… 657	*Aeromonas*
*qnrS6*^a^	657	HQ631376	1… 657	*Aeromonas*
*qnrS7*–*qnrS8* not public yet				

* Last update: July 8th 2012. According to http://www.lahey.org/qnrStudies

a and nucleotide BLAST searches.

The most common mechanism of MLS_B_ resistance is due to the presence of rRNA methylases, encoded by the *erm* genes. These enzymes methylate the adenine residue(s) resulting in MLS_B_ resistance. The methylated adenine(s) prevents the drugs from binding to the 50S ribosomal subunit. The other two mechanisms efflux and enzymatic inactivation result in resistance to only 1 or 2 classes of antibiotics belonging to the MLS group.

There are currently 77 MLS resistance genes recognized. A new MLS gene must have <79% amino acid identity with all previously characterized MLS genes before receiving a unique name (Roberts et al., 1999; Roberts, 2008). For an actual list of the MLS acquired resistance genes we refer to the website of Dr. Marilyn Roberts, http://faculty.washington.edu/marilynr/.

In addition to the subsection of QUINOLONES currently five families of *qnr* genes have been reported; *qnrA* (7 subtypes), *qnrB* (59 subtypes), *qnrC* (1 subtype), *qnrD* (1 subtype), and *qnrS* (8 subtypes) (Jacoby et al., 2008; Cattoir and Nordmann, 2009; Cavaco et al., 2009; Strahilevitz et al., 2009; Torpdahl et al., 2009).

Another mechanism of conferring resistance to quinolones is represented by the plasmid-borne gene *qepA*, which codes for an efflux pump that can export hydrophilic fluoroquinolones, e.g., ciprofloxacin and enrofloxacin (Périchon et al., 2007; Yamane et al., 2007). A variant of this resistance pump, QepA2, was identified in an *E. coli* isolate from France (Cattoir et al., 2008).

Regarding TETRACYCLINE, *Resistance mechanisms*, currently there are 45 different acquired tetracycline resistance determinants recognized (Roberts, 1996, 2005; Brown et al., 2008) (Table 6). For an up-to-date list of the acquired tetracycline resistance genes, we refer to the website of Dr. Marilyn Roberts, http://faculty.washington.edu/marilynr/. Among these, 26 of the *tet* genes, 2 of the *otr* genes and the only *tcr* determinant code for efflux pumps, whereas 11 *tet* genes and 1 *otr* gene code for ribosomal protection proteins (RPPs). The enzymatic inactivation mechanism can be attributed to 3 *tet* genes. The *tet*(U) determinant represents an unknown tetracycline resistance mechanism since its sequence does not appear to be related to either efflux or RPPs, nor to the inactivation enzymes. The efflux and RPP encoding genes are found in members of Gram-positive, Gram-negative, aerobic, as well as anaerobic bacteria. In contrast, the enzymatic tetracycline inactivation mechanism has so far only been identified in Gram-negative bacteria. The *tet*(M) has the broadest host range of all tetracycline resistance genes, whereas *tet*(B) gene has the widest range among the Gram-negative bacteria. In recent years published data indicate that there are increasing numbers of Gram-negative bacteria that carry *tet* genes originally identified in Gram-positive bacteria (Roberts, 2002).

**Table 6:** Acquired tetracycline resistance genes^*^.

**Mechanism**	**Gene**	**Length (nt)**	**Accession number**	**Coding region**	**Genera**
Efflux	*otr*(B)	1692	AF079900	40… 1731	*Mycobacterium*, *Streptomyces*
	*otr*(C)	1056	AY509111	324… 1379	*Streptomyces*
	*tcr*	1539	D38215	516… 2054	*Streptomyces*
	*tet*(A)	1200	X00006	1328… 2527	*Acinetobacter*, *Aeromonas*, *Alcaligenes, Bordetella*, *Chryseobacterium*, *Citrobacter*, *Edwardsiella*, *Enterobacter*, *Escherichia*, *Flavobacterium*, *Klebsiella*, *Laribacter*, *Plesiomonas*, *Proteus*, *Pseudomonas*, *Salmonella*, *Serratia*, *Shigella*, *Variovorax*, *Veillonella*, *Vibrio*
	*tetA*(P)	1263	L20800	1063… 2325	*Clostridium*
	*tet*(B)	1206	J01830	1608… 2813	*Acinetobacter*, *Actinobacillus*, *Aeromonas*, *Aggregatibacter*, *Brevundimonas*, *Citrobacter*, *Enterobacter*, *Erwinia*, *Escherichia*, *Haemophilus*, *Klebsiella*, *Mannheimia*, *Moraxella*, *Neisseria*, *Pantoea*, *Pasteurella*, *Photobacterium*, *Plesiomonas*, *Proteus*, *Providencia*, *Pseudomonas*, *Roseobacter*, *Salmonella*, *Serratia*, *Shigella*, *Treponema*, *Vibrio*, *Yersinia*
	*tet*(C)	1191	X01654	86… 1276	*Aeromonas*, *Bordetella*, *Chlamydia*, *Citrobacter*, *Enterobacter*, *Escherichia*, *Francisella*, *Halomonas*, *Klebsiella*, *Proteus*, *Pseudomonas*, *Roseobacter*, *Salmonella*, *Serratia*, *Shigella*, *Vibrio*
	*tet*(D)	1185	X65876	1521… 2705	*Aeromonas*, *Alteromoas*, *Citrobacter*, *Edwardsiella*, *Enterobacter*, *Escherichia*, *Halomonas*, *Klebsiella*, *Morganella*, *Pasteurella*, *Photobacterium*, *Proteus*, *Salmonella*, *Shewanella*, *Shigella*, *Vibrio*, *Yersinia*
	*tet*(E)	1218	L06940	21… 1238	*Aeromonas*, *Alcaligenes*, *Escherichia*, *Flavobacterium*, *Plesiomonas*, *Proteus*, *Providencia*, *Pseudomonas*, *Roseobacter*, *Serratia*, *Vibrio*
	*tet*(G)	1128	AF071555	6644… 7771	*Acinetobacter*, *Brevundimonsa*, *Escherichia*, *Fusobacterium*, *Mannheimia*, *Ochrobactrum*, *Pasteurella*, *Proteus*, *Providencia*, *Pseudomonas*, *Roseobacter*, *Salmonella*, *Shewanella*, *Vibrio*
	*tet*(H)	1203	U00792	716… 1918	*Acinetobacter*, *Actinobacillus*, *Histophilus, Mannheimia*, *Moraxella*, *Pasteurella, Psychrobacter*
	*tet*(J)	1197	AF038993	1084… 2280	*Escherichia*, *Morganella*, *Proteus*
	*tet*(K)	1380	M16217	305… 1684	*Bacillus*, *Clostridium*, *Enterococcus*, *Eubacterium*, *Haemophilus*, *Lactobacillus*, *Listeria*, *Mycobacterium*, *Nocardia*, *Peptostreptococcus*, *Staphylococcus*, *Streptococcus*, *Streptomyces*
	*tet*(L)	1377	D00006	189… 1565	*Acinetobacter*, *Actinobacillus*, *Actinomyces*, *Bacillus*, *Bifidobacterium*, *Citrobacter*, *Clostridium*, *Enterobacter*, *Enterococcus*, *Escherichia*, *Flavobacterium*, *Fusobacterium*, *Geobacillus*, *Kurthia*, *Lactobacillus*, *Listeria*, *Mannheimia*, *Morganella*, *Mycobacterium*, *Nocardia*, *Ochrobactrum*, *Oceanobacillus, Paenibacillus*, *Pasteurella*, *Pediococcus*, *Peptostreptococcus*, *Proteus*, *Pseudomonas*, *Rahnella*, *Salmonella*, *Sporosarcina*, *Staphylococcus*, *Streptococcus*, *Streptomyces*, *Variovorax*, *Veillonella*, *Virgibacillus*
	*tet*(V)	1260	AF030344	462… 1721	*Mycobacterium*
	*tet*(Y)	1176	AF070999	1680… 2855	*Aeromonas*, *Escherichia*, *Photobacterium*
	*tet*(Z)	1155	AF121000	11880… 13034	*Corynebacterium*, *Lactobacillus*
	*tet*(30)	1185	AF090987	1130… 2314	*Agrobacterium*
	*tet*(31)	1233	AJ250203	1651… 2883	*Aeromonas, Gallibacterium*
	*tet*(33)	1224	AJ420072	22940… 24163	*Arthrobacter, Corynebacterium*
	*tet*(35)	1110	AF353562	2213… 3322	*Stenotrophomonas*, *Vibrio*
	*tet*(38)	1353	AY825285	1… 1353	*Staphylococcus*
	*tet*(39)	1188	AY743590	749… 1936	*Acinetobacter, Alcaligenes, Brevundimonas, Enterobacter*, *Providencia, Stenotrophomonas*
	*tet*(40)	1221	AM419751	14211… 15431	*Clostridium*
	*tet*(41)	1182	AY264780	1825… 3006	*Serratia*
	*tet*(42)	1287	EU523697	687… 1973	*Bacillus*, *Microbacterium*, *Micrococcus*, *Paenibacillus*, *Pseudomonas*, *Staphylococcus*
	*tet*(43)	1560	GQ244501	60… 1619	Uncultured
Enzymatic	*tet*(X)	1167	M37699	586… 1752	*Bacteroides*, *Pseudomonas, Sphingobacterium*
	*tet*(34)	465	AB061440	306… 770	*Aeromonas*, *Pseudomonas*, *Serratia*
	*tet*(37)	327	AF540889	1… 327	Uncultured
Ribosomal protection	*otr*(A)	1992	X53401	349… 2340	*Bacillus, Mycobacterium*, *Streptomyces*
	*tet*B(P)	1959	L20800	2309… 4267	*Clostridium*
	*tet*(M)	1920	U08812	1981… 3900	*Abiotrophia*, *Acinetobacter*, *Actinomyces*, *Aerococcus*, *Aeromonas*, *Afipia*, *Arthrobacter*, *Bacillus*, *Bacterionema*, *Bacteroides*, *Bifidobacterium*, *Brachybacterium, Catenibacterium*, *Clostridium*, *Corynebacterium*, *Edwardsiella*, *Eikenella*, *Enterobacter*, *Enterococcus*, *Erysipelothrix*, *Escherichia*, *Eubacterium*, *Flavobacterium*, *Fusobacterium*, *Gardnerella*, *Gemella*, *Granulicatella*, *Haemophilus*, *Kingella, Klebsiella*, *Kurthia*, *Lactobacillus*, *Lactococcus, Listeria*, *Microbacterium*, *Mycoplasma*, *Neisseria*, *Paenibacillus*, *Pantoea*, *Pasteurella*, *Peptostreptococcus*, *Photobacterium*, *Prevotella*, *Pseudoalteromonas, Pseudomonas*, *Ralstonia*, *Selenomonas*, *Serratia*, *Shewanella, Staphylococcus*, *Streptococcus*, *Streptomyces*, *Ureaplasma*, *Veillonella*, *Vibrio*
	*tet*(O)	1920	M18896	207… 2126	*Actinobacillus*, *Aerococcus*, *Anaerovibrio*, *Bifidobacterium*, *Butyrivibrio*, *Campylobacter*, *Clostridium*, *Enterococcus*, *Eubacterium*, *Fusobacterium*, *Gemella*, *Lactobacillus*, *Megasphaera*, *Mobiluncus*, *Neisseria*, *Peptostreptococcus*, *Psychrobacter*, *Staphylococcus*, *Streptococcus*
	*tet*(Q)	1926	Z21523	362… 2287	*Anaerovibrio*, *Bacteroides*, *Capnocytophaga*, *Clostridium*, *Eubacterium*, *Fusobacterium*, *Gardnerella*, *Lactobacillus*, *Mitsuokella*, *Mobiluncus*, *Neisseria*, *Peptostreptococcus*, *Porphyromonas*, *Prevotella*, *Ruminococcus*, *Selenomonas*, *Streptococcus*, *Subdolgranulum*, *Veillonella*
	*tet*(S)	1926	L09756	447… 2372	*Enterococcus*, *Lactobacillus*, *Lactococcus, Listeria*, *Staphylococcus*, *Streptococcus*, *Veillonella*
	*tet*(T)	1956	L42544	478… 2433	*Lactobacillus*, *Streptococcus*
	*tet*(W)	1920	AJ222769	3687… 5606	*Acidaminococcus*, *Actinomyces*, *Arcanobacterium*, *Bacillus*, *Bacteroides*, *Bifidobacterium*, *Butyrivibrio*, *Clostridium*, *Fusobacterium*, *Lactobacillus*, *Megasphaera*, *Mitsuokella*, *Neisseria*, *Porphyromonas*, *Prevotella*, *Roseburia*, *Selenomonas*, *Staphylococcus*, *Streptococcus*, *Streptomyces*, *Subdolgranulum*, *Veillonella*
	*tet*(32)	1920	DQ647324	181… 2100	*Eubacterium*, *Streptococcus*
	*tet*(36)	1923	AJ514254	2534… 4456	*Bacteroides*, *Clostridium*, *Lactobacillus*
	*tet*(44)	1923	FN594949	25245… 27167	*Campylobacter, Clostricium*
	*tet*	1920	M74049	343… 2261	*Streptomyces*
Unknown	*tet*(U)	318	U01917	413… 730	*Enterococcus*, *Staphylococcus*, *Streptococcus*

* Last update: January 6th 2012. Adapted from http://faculty.washington.edu/marilynr/. The efflux genes tet(45) and tet(46) have been named but not yet published.

To the subsection TRIMETHOPRIM, *Resistance mechanisms*. Initially, the acquired DHFRs fell into two distinct families A and B, encoded by the *dfrA* and *dfrB* genes (Howell, 2005). Up to now 6 plasmid-mediated families can be distinguished with relatively few *dfr* determinants originating from Gram-positive bacteria (Table 7). The *dfrK* and *dfrA28* genes are the newest additions to the trimethoprim resistance determinant family (Kadlec and Schwarz, 2009; Kadlec et al., 2011). In contrast to the latest reported DHFRs, the oldest families, *dfrA* and *dfrB*, each contain several members (Roberts, 2002; Levings et al., 2006). For example, the *dfrA* group accomodates over 30 published genes; however, unpublished, *dfrA* variants are also present in the public DNA libraries and some genes apparently have changed nomenclature (Table 7).

**Table 7:** Acquired trimethoprim resistance genes^*^.

**Gene**	**Sub-family**	**Gene(s) included**	**Length (nt)**	**Accession number**	**Coding region**	**Genera**
*dfrA1*	*dfrA1*-group	*dhfrIb*, *dfr1*, *dhfrI*	474	X00926	236… 709	*Actinobacter*, *Enterobacter*, *Escherichia*, *Klebsiella*, *Laribacter*, *Morganella*, *Pasteurella*, *Proteus*, *Pseudomonas*, *Salmonella, Serratia*, *Shigella*, *Vibrio*
*dfrA3*	−	−	489	J03306	103… 591	*Salmonella*
*dfrA5*	*dfrA1*-group	*dhfrV*, *dfrV*	474	X12868	1306… 1779	*Actinobacter*, *Aeromonas*, *Comomonas, Enterobacter*, *Escherichia*, *Klebsiella*, *Pseudomonas*, *Salmonella, Vibrio*
*dfrA6*	*dfrA1*-group	*dfrVI*	474	Z86002	336… 809	*Proteus*
*dfrA7*	*dfrA1*-group	*dhfrVII*, *dfrVII*, *dfrA17*	474	X58425	594… 1067	*Actinobacter*, *Escherichia*, *Proteus*, *Salmonella*, *Shigella*
*dfrA8*	−	−	510	U10186	711… 1220	*Escherichia*
*dfrA9*	−	−	534	X57730	726… 1259	*Escherichia*
*dfrA10*	−	−	564	L06418	5494… 6057	*Actinobacter*, *Escherichia*, *Klebsiella*, *Salmonella*
*dfrA12*	*dfrA12*-group	*dhfrXII*, *dfr12*	498	Z21672	310… 807	*Actinobacter*, *Aeromonas*, *Citrobacter*, *Edwardsiella*, *Enterobacter*, *Escherichia*, *Klebsiella*, *Proteus*, *Providencia*, *Pseudomonas*, *Serratia*, *Salmonella, Staphylococcus*, *Stenotrophomonas*
*dfrA13*	*dfrA12*-group	−	498	Z50802	718… 1215	*Escherichia*
*dfrA14*	*dfrA1*-group	*dhfrIb*	474	Z50805	72… 545	*Achromobacter*, *Aeromonas*, *Escherichia*, *Klebsiella*, *Salmonella, Vibrio*
*dfrA15*	*dfrA1*-group	*dhfrXVb*	474	Z83311	357… 830	*Actinobacter*, *Enterobacter*, *Escherichia*, *Klebsiella*, *Morganella*, *Proteus*, *Pseudomonas*, *Vibrio*
*dfrA16*	*dfrA1*-group	*dhfrXVI*, *dfr16*	474	AF174129	1352… 1825	*Aeromonas*, *Escherichia*, *Klebsiella*, *Salmonella*
*dfrA17*	*dfrA1*-group	*dhfrXVII*, *dfr17*	474	AB126604	98… 571	*Actinobacter*, *Enterobacter*, *Escherichia*, *Klebsiella*, *Kluyvera*, *Laribacter*, *Pseudomonas*, *Salmonella, Serratia*, *Shigella*, *Staphylococcus, Stenotrophomonas*
*dfrA18*	−	*dfrA19*	570	AJ310778	7004… 7573	*Enterobacter*, *Klebsiella*, *Salmonella*
*dfrA20*	−	−	510	AJ605332	1304… 1813	*Pasteurella*
*dfrA21*	*dfrA12*-group	*dfrxiii*	498	AY552589	1… 498	*Escherichia*, *Klebsiella*, *Salmonella*
*dfrA22*	*dfrA12*-group	*dfr22*, *dfr23*	498	AJ628423	325… 822	*Escherichia*, *Klebsiella*, *Serratia*
*dfrA23*	−	−	561	AJ746361	6743… 7303	*Salmonella*
*dfrA24*	−	−	558	AJ972619	83… 640	*Escherichia*
*dfrA25*	*dfrA1*-group	−	459	DQ267940	54… 512	*Citrobacter*, *Klebsiella*, *Salmonella*, *Serratia*
*dfrA26*	−	–	552	AM403715	303… 854	*Escherichia*
*dfrA27*	*dfrA1*-group	*dfr*	474	EU675686	2543… 3016	*Aeromonas*, *Escherichia*, *Klebsiella*, *Serratia*, *Vibrio*
*dfrA28*	*dfrA1*-group	−	474	FM877476	116… 589	*Aeromonas*
*dfrA29*	−	*dfrVII*, *dfrA7*	472	AM237806	615… 1086	*Salmonella*
*dfrA30*	−	*dhfrV*	474	AM997279	705… 1178	*Klebsiella*
*dfrA31*	−	*dfr6*	474	AB200915	1832… 2305	*Escherichia*, *Vibrio*
*dfrA32*	*dfrA1*-group	*−*	474	GU067642	535… 1008	*Laribacter*, *Salmonella*
*dfrA33*	*dfrA12*-group	*−*	498	FM957884	88… 585	Unknown
*dfrB1*	−	*dhfrIIa*, *dfr2a*	237	U36276	717… 953	*Aeromonas*, *Bordetella*, *Escherichia*, *Klebsiella, Pseudomonas*
*dfrB2*	−	*dhfrIIb*, *dfr2b*	237	J01773	809… 1045	*Escherichia*
*dfrB3*	−	*dhfrIIc*, *dfr2c*	237	X72585	5957… 6193	*Aeromonas*, *Enterobacter*, *Escherichia*, *Klebsiella*
*dfrB4*	−	*dfr2d*	237	AJ429132	69… 305	*Aeromonas*, *Escherichia*, *Klebsiella*
*dfrB5*	−	*dfr2e*	237	AY943084	2856… 3092	*Pseudomonas*
*dfrB6*	−	−	237	DQ274503	394… 630	*Salmonella*
*dfrB7*	−	*−*	237	DQ993182	244… 480	*Aeromonas*
*dfrB8*	−	*−*	249	GU295656	1048… 1296	*Aeromonas*
*dfrD*	−	−	489	Z50141	94… 582	*Listeria*, *Staphylococcus*
*dfrG*	−	*−*	498	AB205645	1013… 1510	*Enterococcus*, *Staphylococcus*
*dfrK*		−	492	FM207105	2788… 3279	*Enterococcus, Staphylococcus*

* Last update: December 12th 2011. Partly adapted from Grape (2006), Partridge et al. (2009), and nucleotide BLAST searches.

Furthermore, we suggest an additional section concerning oxazolidinones.

## Oxazolidinones

### History and action mechanism

Linezolid is to date the only FDA-approved oxazolidinone (Shaw and Barbachyn, 2011). It was approved in 2000 for the treatment of serious infections caused by Gram-positive bacteria resistant to other antibiotics, such as vancomycin-resistant enterococci (VRE) and methicillin-resistant *Staphylococcus aureus* (MRSA) (Long and Vester, 2012). As such linezolid is considered one of the last resort antimicrobial agents in human medicine. It has not been approved for use in veterinary medicine. Oxazolidinones bind at the P site of the ribosome and inhibit the formation of the initiation complex, which consists of mRNA, f-Met tRNA, and the 50S ribosomal subunit (Shaw and Barbachyn, 2011; Long and Vester, 2012).

### Resistance mechanism

Various mutations located in the peptidyl transferase loop of domain V of 23S rRNA as well as mutations in the genes for the ribosomal proteins L3 and L4, all associated with resistance to oxazolidinones, have been identified (reviewed by Long and Vester, 2012). A single gene, *cfr*, has been identified to confer transferable resistance to oxazolidinones. This gene codes for a methyltransferase that targets A2503 in 23S rRNA (Kehrenberg et al., 2005). Besides oxazolidinone resistance, it also confers resistance to phenicols, lincosamides, pleuromutilins, and streptogramin A antibiotics. Although initially identified in coagulase-negative staphylococci of animal origin, the gene *cfr* has now been detected in a wide variety of staphylococci of human and animal origin, including a Panton-Valentin leukocidin-positive MRSA USA300 (Shore et al., 2010) and livestock-associated MRSA ST398 (Kehrenberg et al., 2009). More recently, the *cfr* gene has also been identified in *Bacillus* spp. (Dai et al., 2010) and *Enterococcus faecalis* (Liu et al., 2012), but also in Gram-negative bacteria, such as *Proteus vulgaris* (Wang et al., 2011) and *Escherichia. coli* (Wang et al., 2012). Plasmids and insertion sequences seem to play an important role in the spread of this gene across species and genus boundaries.
